# miRNA dysregulation and the risk of metastasis and invasion in papillary thyroid cancer: a systematic review and meta-analysis

**DOI:** 10.18632/oncotarget.16681

**Published:** 2017-03-29

**Authors:** Tiantian Wang, Hao Xu, Ming Qi, Sheng Yan, Xingsong Tian

**Affiliations:** ^1^ Department of Breast and Thyroid Surgery, Shandong Provincial Hospital Affiliated to Shandong University, Jinan, Shandong, China; ^2^ Department of Neurosurgery, The First Hospital Affiliated to Sun Yat-sen University, Guangzhou, Guangdong, China

**Keywords:** miRNA, invasive thyroid cancer, metastasis, meta-analysis

## Abstract

Thyroid cancer (TC) is the most common endocrine malignancy, with an incidence continuing to grow every year. Although thyroid cancer as a whole is generally indolent and relatively easy to treat, some subtypes carry a higher rate of metastasis and cancer-related mortality. A growing number of studies have focused on the dysregulation of miRNAs in TC. However, differences in methods make comparison of gene profiling data difficult. A meta-analysis of published studies comparing miRNA expression data of invasive thyroid carcinoma with paired non-invasive tumors or normal tissues was performed by searching the literature for “invasion”, “thyroid cancer”, and “miRNA”. This revealed 29 dysregulated miRNAs associated with TC in 16 articles; the presence of invasion was confirmed in each respective article by laboratory research or patient follow-up. Among these miRNAs, miRNA-146b, miRNA-221, and miRNA-222 were analyzed further due to their higher frequencies across multiple studies. Of these studies, 6 were included in the meta-analysis, as they compared invasive PTC with paired normal tissues or non-invasive PTC.

## INTRODUCTION

Thyroid carcinoma (TC) is the most common endocrine malignancy, the incidence was increasing year by year. Papillary thyroid cancer(PTC) takes up more than 80% of TC. The overall survival rate of patients with papillary thyroid carcinoma is high [1], however, increased cancer recurrence and cancer-related mortality are noted in a portion of patients with papillary thyroid carcinoma [2]. Distinguishing these tumors from classic tumors has therefore become a hot topic of research in recent years.

MicroRNAs (miRNAs) are a recently identified class of small, endogenous, non-coding RNAs that act as negative regulators of gene expression [3]. miRNAs are abundant and ubiquitous, and impact almost all fundamental cell processes such as growth, differentiation, apoptosis, and adhesion [4]. Dysregulation of miRNA expression is a common feature in many types of human cancers, including thyroid cancer [5 6].

## RESULTS

### Study collection and inclusion

We initially collected 106 studies using the described strategy. 84 articles were excluded because they were not relevant to the aim of our study. 29 miRNAs in 16 articles were subsequently identified [7-23], with the function of dysregulated miRNAs confirmed through patient follow-up or other laboratory experiments. The dysregulated miRNAs are shown in detail in Tables 1a-1b.

**Table 1a:** Studies demonstrating an association between upregulated miRNAs and invasion

miRNA	Author	Year	Country	Method
MiRNA-146b	Chou	2010	China	qRT-PCR
	Chou	2012	China	qRT-PCR
	Deng	2015	China	qRT-PCR
	Lee	2013	Australia	Micro array
	Lima	2016	Brazil	qRT-PCR
	Yang	2013	China	Micro array
	Wang	2013	China	Micro array
MiRNA-222	Chou	2010	China	qRT-PCR
	Lee	2013	Australia	Micro array
	Jikuzono	2013	Africa	qRT-PCR
	Yang	2013	China	Micro array
	Chou	2012	China	qRT-PCR
	Wang	2013	China	Micro array
MiRNA-221	Chou	2010	China	qRT-PCR
	Chou	2012	China	qRT-PCR
	Jikuzono	2013	Africa	qRT-PCR
	Wang	2013	China	Micro array
	Zhou	2012	China	Northern blot
	Yang	2013	China	Micro array
MiRNA-4295	Shao	2015	China	Micro array
MiRNA-101	Wang	2014	China	qRT-PCR
MiRNA-183	Wei	2015	China	qRT-PCR
MiRNA-210	Yang	2013	China	Micro array
MiRNA-584	Xiang	2015	China	qRT-PCR
MiRNA-1244	Yang	2013	China	Micro array
MiRNA-134	Yang	2013	China	Micro array
MiRNA-214	Yang	2013	China	Micro array
MiRNA-1202	Wang	2013	China	Micro array
MiRNA-193	Wang	2013	China	Micro array
MiRNA-2861	Wang	2013	China	Micro array

**Table 1b:** Studies demonstrating an association between downregulated miRNAs and invasion

MiRNA	Author	Year	Country	Method
MiRNA-539	GU	2015	China	qRT-PCR
MiRNA-144	Guan	2014	China	qRT-PCR
MiRNA-7	Hua	2016	China	Micro array
	Wang	2013	China	Micro array
MiRNA-182	Zhu	2014	China	Micro array
MiRNA-126	Xiong	2015	USA	qRT-PCR
MiRNA-486	Yang	2013	China	Micro array
MiRNA-206	Zhang	2015	China	Micro array
MiRNA-1302	Yang	2013	China	Micro array
MiRNA-1231	Yang	2013	China	Micro array
MiRNA-637	Yang	2013	China	Micro array
MiRNA-1826	Yang	2013	China	Micro array
MiRNA-1225	Yang	2013	China	Micro array
MiRNA-564	Wang	2013	China	Micro array
MiRNA-664	Wang	2013	China	Micro array
MiRNA-542	Wang	2013	China	Micro array

### miRNA inclusion

Among these miRNAs, miRNA-146b, miRNA-221, and miRNA-222 were analyzed further due to their appearance at higher frequency. Of the studies that analyzed these miRNAs, 6 were selected for meta-analysis because they compared invasive PTC with paired normal tissues or non-invasive PTC [7-12]. These dysregulated miRNAs are shown in detail in Table 2.

**Table 2:** Studies included in the meta-analysis

MiRNA	Author	Year	Country	Methods	Samples (tumor/control)
MiRNA-146b	Chou	2010	China	qRT-PCR	16/16
	Chou	2012	China	qRT-PCR	30/41
	Deng	2015	China	qRT-PCR	30/30
	Lee	2013	Australia	Micro array	9/17
	Yang	2013	China	Micro array	3/3
	Wang	2013	China	Micro array	3/3
MiRNA-221	Chou	2010	China	qRT-PCR	16/16
	Chou	2012	China	qRT-PCR	30/41
	Lee	2013	Australia	Micro array	9/17
	Yang	2013	China	Micro array	3/3
MiRNA-222	Chou	2010	China	qRT-PCR	16/16
	Chou	2012	China	qRT-PCR	30/41
	Lee	2013	Australia	Micro array	9/17
	Yang	2013	China	Micro array	3/3

### Data characteristics

The 6 included articles are shown in Table 2. In all, 201 samples were analyzed, including 91 invasive tumors and 110 paired control tissues (either non-invasive tumors or normal tissue).

### Meta-analysis results

miRNA-146b was upregulated in all 6 studies, while miRNA-221 and miRNA-222 were upregulated in 4 studies. The forest plot is shown in Figure 3. Given that I^2^ > 50%, the fixed-effects model was used. *p* < 0.00001 for each miRNA was determined, suggesting that miRNA-146b, miRNA-221, and miRNA 222 are all upregulated in invasive tumors compared with paired control.

**Figure 1:**
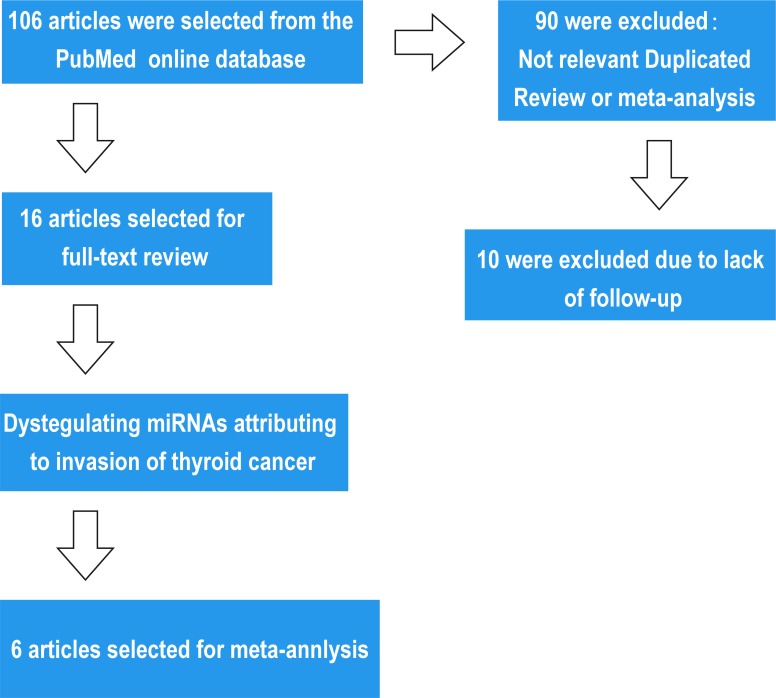
Flow chart of search strategy

**Figure 2:**
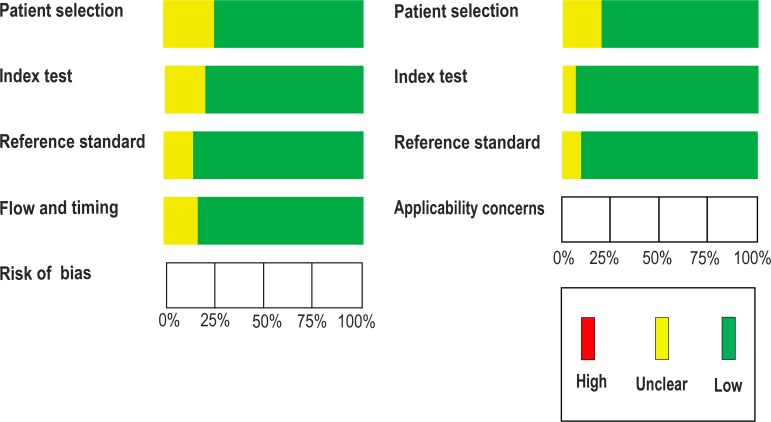
Quality of selected studies according to QUADAS-2 guidelines

**Figure 3:**
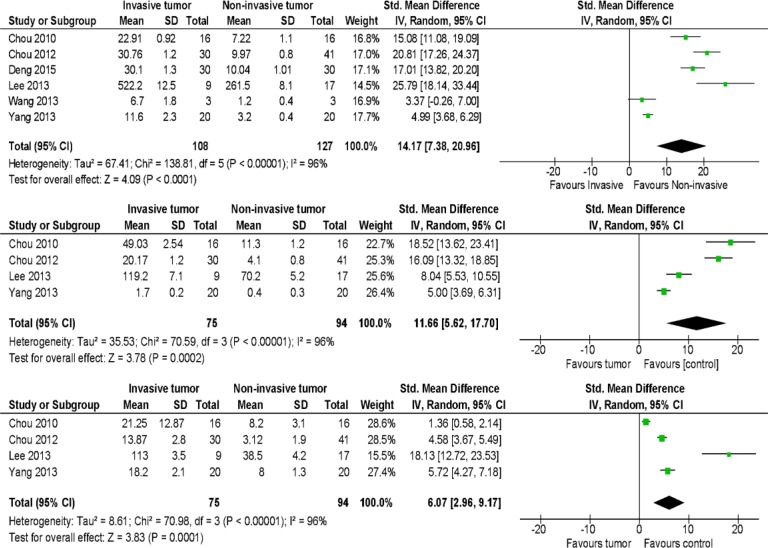
Forest plots for miRNA-146, miRNA-221, and miRNA-222 in the described articles As I^2^ > 50%, a random-effects model was used. *p* < 0.05, indicated that miRNA-146, miRNA-221, and miRNA-222 were all upregulated in invasive tumors compared with paired controls.

### Publication bias

**Figure 4:**
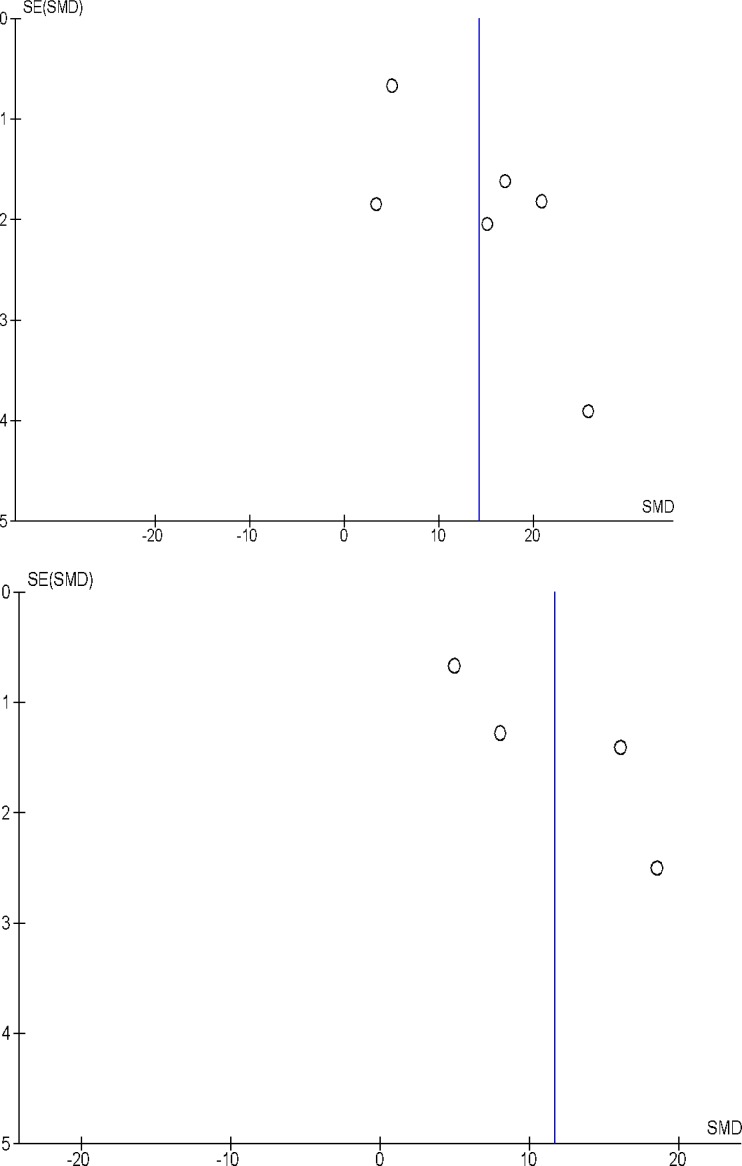

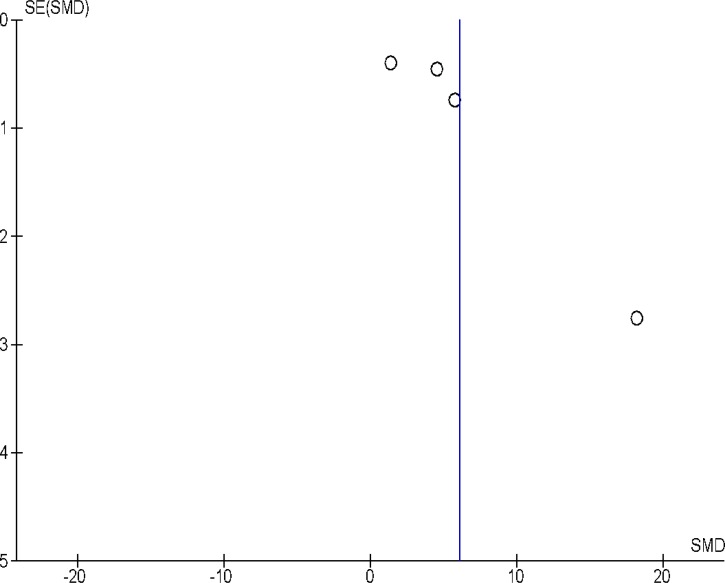
Funnel plot to measure publication bias, with regards to miRNA-146 Due to the relative lack of research studies, publication bias is somewhat unavoidable.

Publication bias in our study was assessed using funnel plot analysis. As shown in Figure 4, there was minimal publication bias in this research, though some amount is unavoidable due to the lack of prior research.

## DISCUSSION

Thyroid carcinoma (TC) is the most common endocrine malignancy. The overall survival rate of patients with thyroid carcinoma is high [1], however, increased cancer recurrence and cancer-related mortality are noted in a portion of patients with papillary thyroid carcinoma [2]. Distinguishing these tumors from classic non-lethal papillary tumors has therefore become a hot topic of research in recent years.

MicroRNAs (miRNAs) are a recently identified class of small, endogenous, non-coding RNAs that act as negative regulators of gene expression [3]. miRNAs are abundant and ubiquitous, and they impact almost all fundamental cell processes including growth, differentiation, apoptosis, and adhesion [4]. Dysregulation of miRNA expression is a common feature of many types of human cancers, including thyroid cancer [5 6].

It is well-known that many dysregulated miRNAs contribute to the tumorigenesis and progression of tumors. Many studies show a direct relationship between dysregulated miRNAs and the molecular mechanisms they induce to affect tumor invasion, but there has been less research addressing the clinical features surrounding invasion ability. In this article, we explored the relationship between dysregulated miRNAs and invasion ability. We searched the PubMed online database using the keywords “papillary thyroid carcinoma” or “thyroid carcinoma”, “miRNA”, and “invasion” and collected 106 records following the strategy described above. 84 articles were excluded because they were not relevant to the aim of our study. 29 miRNAs in 16 articles were identified with the function of the dysregulated miRNAs confirmed via patient follow-up or other laboratory experiments. Among these miRNAs, miRNA-146b, miRNA-221, and miRNA-222 were analyzed further. In total, 6 studies were included in the meta-analysis because they compared invasive PTC with paired normal tissues or non-invasive PTC.

The present meta-analysis has several limitations. Firstly, due to the lack of significant current research, only 6 articles comparing invasive PTC with paired non-invasive PTC or normal tissues were able to be included. Secondly, the divergence of incorporated studies also likely contributed to the statistical differences. Finally, there was a lack of strong correlation between overall patient survival statistics and the presence of dysregulated miRNAs due to lack of patient follow-up.

## MATERIALS AND METHODS

### Search strategy

A search for dysregulated miRNAs in papillary thyroid carcinoma was performed by querying the PubMed online database with the terms “miRNA” or “microRNA” or “miR”, “thyroid carcinoma” or “papillary thyroid carcinoma”, and “invasion”. Titles and abstracts of the obtained articles were screened, and full texts of the articles of interest were further evaluated.

### Inclusion and exclusion criteria

Research studies were considered to be eligible if they met the following criteria: (1) the study focused on patients with any type of papillary thyroid carcinoma; (2) the effect of the dysregulated miRNA on invasion was confirmed via analysis of the patients’ metastases or other experimental tests in the same article; (3) for meta-analysis articles, miRNA effect was confirmed through patient follow-up. Articles were excluded based on the following criteria: (1) reviews, letters, comments, or pure laboratory studies; (2) absence of specific evidence showing the dysregulated miRNA had any influence on invasion; (3) discrepant conclusions across different articles.

### Statistical analysis

The fixed-effects and random-effects models were used for the meta-analysis according to heterogeneity among the pooled studies. The heterogeneity test for the pooled odds ratio (OR) was verified using the I^2^ statistic. A random-effects model was applied if the heterogeneity was significant (*p* < 0.01 or I^2^ > 50%), while a fixed-effects model was applied if the heterogeneity was not significant (*p* > 0.01 or I^2^ < 50%). Publication bias was estimated using a funnel plot. All *p* values were calculated using a two-sided test; differences were considered statistically significant when *p* < 0.05. All statistical analyses were conducted using the Review Manager 5 (Cochrane Tech, London, UK) and Microsoft Excel (Microsoft Corporation, Seattle, WA, USA) software.

## CONCLUSIONS

Current evidence and the findings of this article suggest, despite the limitations described above, that dysregulation of these miRNAs leads to increased invasion ability of thyroid carcinoma.
